# Serotonin-Secreting Neuroendocrine Tumours of the Pancreas

**DOI:** 10.3390/jcm9051363

**Published:** 2020-05-06

**Authors:** Anna Caterina Milanetto, Matteo Fassan, Alina David, Claudio Pasquali

**Affiliations:** 1 Pancreatic and Endocrine Digestive Surgical Unit, Department of Surgery, Oncology and Gastroenterology, University of Padua, via Giustiniani, 2-35128 Padua, Italy; alina.david@studenti.unipd.it (A.D.); claudio.pasquali@unipd.it (C.P.); 2 Surgical Pathology Unit, Department of Medicine, University of Padua, via Giustiniani, 2-35128 Padua, Italy; matteo.fassan@unipd.it

**Keywords:** pancreatic neuroendocrine neoplasm, primary pancreatic carcinoid, serotonin-secreting pancreatic tumour, serotonin-producing pancreatic tumour

## Abstract

Background: Serotonin-secreting pancreatic neuroendocrine tumours (5-HT-secreting pNETs) are very rare, and characterised by high urinary 5-hydroxyindole-acetic acid (5-HIAA) levels (or high serum 5-HT levels). Methods: Patients with 5-HT-secreting pancreatic neoplasms observed in our unit (1986–2015) were included. Diagnosis was based on urinary 5-HIAA or serum 5-HT levels. Results: Seven patients were enrolled (4 M/3 F), with a median age of 64 (range 38–69) years. Two patients had a carcinoid syndrome. Serum 5-HT was elevated in four patients. Urinary 5-HIAA levels were positive in six patients. The median tumour size was 4.0 (range 2.5–10) cm. All patients showed liver metastases at diagnosis. None underwent resective surgery; lymph node/liver biopsies were taken. Six lesions were well-differentiated tumours and one a poorly differentiated carcinoma (Ki67 range 3.4–70%). All but one patient received chemotherapy. Four patients received somatostatin analogues; three patients underwent ablation of liver metastases. One patient is alive with disease 117 months after observation. All the others died from disease progression after a follow-up within 158 months. Conclusions: Primary 5-HT-secreting pNETs are mostly metastatic to the liver; patients are not amenable to resective surgery. Despite high 5-HIAA urinary levels, few patients present with carcinoid syndrome. A five-year survival rate of 42.9% may be achieved with multimodal treatment.

## 1. Introduction

“Carcinoid tumours” were originally classified into foregut (including those arising in the pancreas), midgut and hindgut tumours, according to their embryologic origin [1]. The term “carcinoid” has been used for a long time to indicate midgut or small intestinal neuroendocrine tumours (NETs), which produce serotonin (5-HT), and cause the typical “carcinoid syndrome”. These NETs can be identified by twenty-four-hour measurement of urinary 5-hydroxyindole-acetic acid (5-HIAA), which has an 88% specificity for 5-HT-producing NETs [2]. 

Pancreatic NETs (pNETs) may show positive immunostaining for hormones, neuropeptides and amines, including 5-HT [3,4], and about 4–8% of small pNETs can show a variable portion of cells staining for 5-HT [5,6], even if they are non-functioning (in which case the patient will not complain of symptoms related to hormonal hypersecretion). Recently, small pNETs with a positive 5-HT staining have been called “serotoninomas” [7], while La Rosa [6] in 2011 proposed the term “5-HT-producing EC cell tumours of the pancreas”.

In the literature, there are different definitions of “pancreatic carcinoids”, and it is not always clear whether reports refer to 5-HT-secreting, or 5-HT-staining pancreatic tumours. The term “foregut carcinoid”, used before the year 2000, includes pNETs with normal levels of 5-HT and urinary 5-HIAA [8], and some pancreatic adenocarcinomas with neuroendocrine differentiation and carcinoid-like symptoms [9,10]. Therefore, it is difficult to estimate the actual incidence and prevalence of 5-HT-secreting tumours of the pancreas.

The aim of the present study is to focus on pancreatic NETs secreting 5-HT, rare entities that may be defined as “5-HT-secreting pNETs” and diagnosed in the presence of a pancreatic mass, an increased urinary 5-HIAA level above the upper limit of normal, and/or an increased serum 5-HT level. The experience of a single high-volume pancreatic and referral centre for NETs is analysed, and the clinic-pathological features, treatment and prognosis of these patients are discussed.

## 2. Methods

Clinical records of patients who were observed for a 5-HT-secreting pNET from January 1986 to December 2015 in our unit were retrieved. The diagnosis of 5-HT-secreting pNET was made in the presence of a pancreatic mass assessed by imaging studies, an increased urinary 5-HIAA level above the upper limit of normal, and/or an increased serum 5-HT level. Patients with only a positive 5-HT immunostaining should be defined as having a non-functioning 5-HT-staining pNET, and were not included in the present study. Patients were diagnosed with a pNET using the following imaging studies: computed tomography-CT scan, magnetic resonance imaging-MRI, 111In-Scintigraphy and/or 18F-FDG positron emission tomography (PET)/CT. Patients presenting with a pNET and a concomitant small intestinal NET, or who had previously been operated on for a small intestinal NET, were not included in the present study. At the time of initial evaluation, plasma and urine samples of the patients were assayed for 5-HT and 5-HIAA, respectively, which since 1992 has been carried out using high-pressure liquid chromatography. Serotonin levels were measured in serum after separation of platelets, apart from the first patient (case n.1), for whom an old radiometric method was used.

The following data were analysed: age, gender, medical history and clinical presentation, blood and urinary tests (serum 5-HT, 24-h urinary 5-HIAA, and other serum tumour markers and gastrointestinal hormones), tumour location in the pancreas, and type of surgery or tumour biopsy. The diagnosis and grading of pancreatic neuroendocrine neoplasms were carried out according to the World Health Organization (WHO) 2019 Classification of Digestive System Tumours [11] and the European Neuroendocrine Tumor Society (ENETS) TNM classification [12]. In particular, tumour size (cm), lymph node metastases (Nx, N0, N1), distant metastases (Mx, M0, M1) and tumour grade (G1, G2 and G3, assessed by mitotic index and Ki67 labelling index) were evaluated. Immunohistochemical analysis was performed for synaptophysin, chromogranin A (CgA) and neuron-specific enolase (NSE), and for expression of other hormones and neuroendocrine markers (insulin, glucagon, somatostatin, pancreatic polypeptide, 5-HT, gastrin, vasoactive intestinal peptide and calcitonin). 

All the patients had a regular follow-up, with clinical evaluation, blood and urinary tests (in particular, serum 5-HT and urinary 5-HIAA) and imaging studies as above (including ^68^Ga-DOTA-peptide PET/CT) to define the extent of the tumour and detect any tumour progression. Other non-surgical treatments, liver metastases embolisation or ablation treatments were recorded. Overall survival (OS) was evaluated in all patients, based on death certificates, or if still living either using a telephone interview or at the last follow-up visit. Follow-up closed in December 2019. Survival curves were estimated using the Kaplan–Meier method. The research was conducted ethically in accordance with the World Medical Association Declaration of Helsinki. Subjects have given their written informed consent to data processing anonymously for research purposes. The ethics committee of the Azienda Ospedaliera di Padova approved the present study (project code: 2872p).

## 3. Results

### 3.1. Patient Characteristics and Laboratory Diagnosis

**Figure 1:**
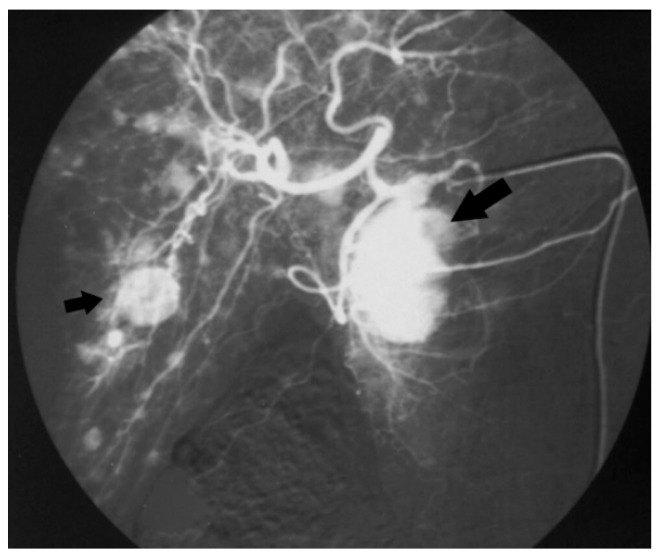
Angiography of the celiac trunk showing a mass in the pancreatic head (big arrow) and multiple liver metastases (small arrow) in a patient with carcinoid syndrome (case n.1).

**Figure 2:**
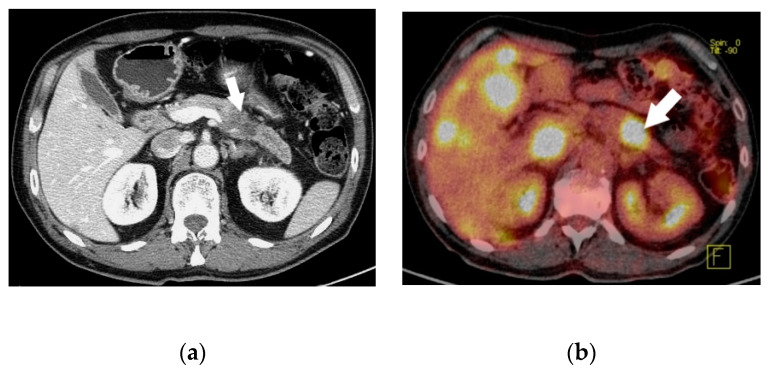
Computed tomography scan 2 (**a**) and ^18^F-FDG positron emission tomography/CT 2 (**b**) showing a pancreatic neuroendocrine tumor in the body of the pancreas (white arrow) with multiple liver metastases (case n.7).

**Figure 3:**
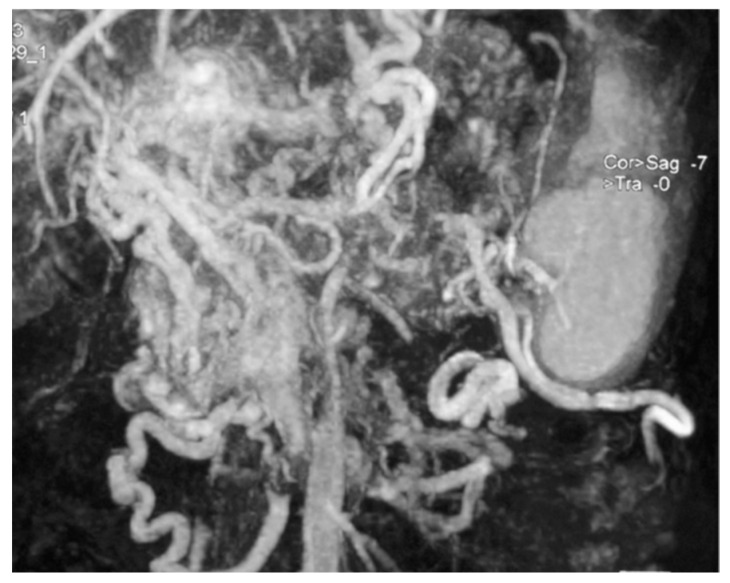
Abdominal magnetic resonance imaging showing several huge mesenteric and left gastric vein compensation collateral circles due to portal vein thrombosis and portal hypertension (case n.6).

Among 239 patients with a histologically confirmed pNET observed in our unit during the study period, seven (2.9%) patients had a 5-HT-secreting pNET. The study population consisted of four men and three women, with a median age of 64 (range 38–69) years. Only two patients had symptoms related to a carcinoid syndrome with flushing and diarrhoea (Figure 1); all the others presented with a non-functioning pNET. The leading presenting symptom was weight loss in three (43%) patients, and two (29%) patients complained about abdominal pain (Figure 2). One patient had a cervical lymphadenopathy, and another presented with jaundice and ascites due to portal vein thrombosis (Figure 3). 

Six patients had increased urinary 5-HIAA levels (up to 18x the upper limit of normal), and four patients had an increased serum 5-HT (up to 5x the upper limit of normal). Notably, four patients with high urinary 5-HIAA levels, two of them also with increased serum 5-HT levels, had no symptoms related to a carcinoid syndrome. Some patients showed a co-secretion of other peptides: four patients had an increased serum CgA, and three had raised calcitonin levels (Table 1).

### 3.2. Histology and Immunohistochemical Features

**Figure 4:**
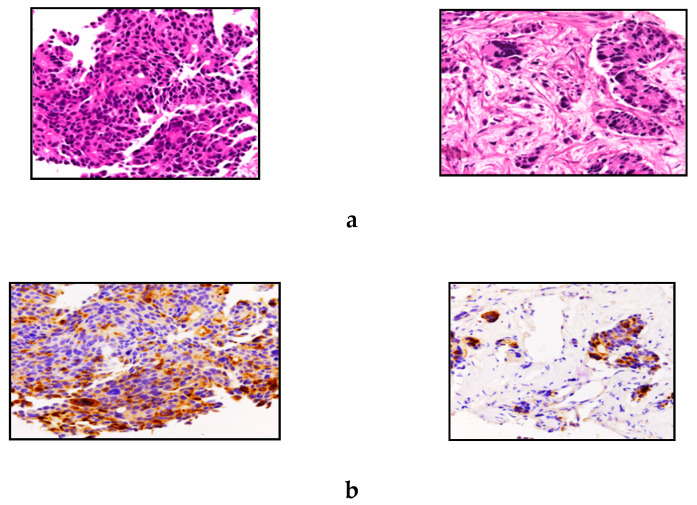
Representative hematoxylin and eosin stain 4 (**a**) and serotonin immunostaining 4 (**b**) of case n.7. The neoplasia was characterized by a trabecular pattern of growth, high mitotic activity (MIB1-labelling index > 70%), areas of necrosis, and high-grade cytonuclear pleomorphism. A final diagnosis of G3 neuroendocrine tumour (NET G3) was reached. The lesion showed a heterogeneous serotonin pattern of staining, which was positive in most neoplastic cells (original magnifications, 20×).

The median primary tumour size was 4.0 (range 2.5–10) cm, and the primary tumour was located in the pancreatic body in four out of seven cases. Despite all patients showing bilobar liver metastases at diagnosis, only two presented with a carcinoid syndrome. None of the patients underwent a pancreatic resection. Therefore, final diagnosis of pNET was made in five patients after a liver biopsy and in the others by lymph node biopsies. Tumour grading (used since 1998) was available in three cases, two G2 NETs (3.4% and 16%) and one G3 NET (70%). Of the other four cases, three were well-differentiated NETs and one was a poorly differentiated large cell neuroendocrine carcinoma (NEC). In particular, the NEC was characterised by a solid growth pattern, large areas of necrosis and large polygonal cells having amphophilic cytoplasm, vesicular chromatin and prominent nucleoli. Data on immunohistochemical analysis were available in five cases, all of them showing a positivity for CgA; in addition, two patients had a positive 5-HT staining (Figure 4). Unfortunately, no remaining tissue was available to perform 5-HT staining, or to detect the other peptides secreted by the tumour.

### 3.3. Prognosis and Follow-Up

All the patients were evaluated in terms of OS, after a median follow-up of 29 (range 5–158) months. Four patients were treated with somatostatin analogues (SS-A), two of whom had a carcinoid syndrome and showed an improvement of symptoms. Three patients also underwent a local ablation of liver metastases (trans-arterial (chemo)-embolisation, microwave ablation), and all but one patient received polychemotherapy regimens. Multimodal treatment with chemotherapy, SS-A and/or loco-regional liver ablation was performed in all the patients, and they demonstrated, occasionally, a long survival (up to 158 months). One patient is still alive with the disease at late evaluation 117 months after diagnosis. All the others died due to disease progression after a median follow-up of 22.5 (range 5–158) months Table 2. Disease-related survival at 1, 3 and 5 years was 71.4%, 42.9% and 42.9%, respectively (Table 2).

## 4. Discussion

The old term “pancreatic carcinoids”, corresponding to 5-HT-producing pNETs, accounts for 0.58% to 1.4% of two large series of “carcinoids” [13,14], but it is difficult to estimate the actual incidence of 5-HT-secreting pNETs, because pNETs causing a clinically evident carcinoid syndrome are very rare [6]. In fact, in a large series of so-called “pancreatic carcinoids”, Soga et al. [14] observed only 23% of patients complaining of a carcinoid syndrome. 

Currently, 5-HT staining and urinary 5-HIAA measurement are not routinely tested or recommended in pNETs [6,7,15]; it is likely that the true incidence and prevalence of 5-HT-producing/secreting pNETs may be underestimated. In our experience, the systematic measurement of urinary 5-HIAA or serum 5-HT in patients with a pNET (although asymptomatic) allowed the selection of a subset of primary pNETs with an excess of 5-HT secretion in the bloodstream, revealed by the excretion of its urinary metabolite 5-HIAA. 

In our series, patients were affected by large pNETs (median size 4 cm, up to 10 cm in size), with multiple liver metastases at presentation. A similar rate of liver metastases (95%) in 22 patients with 5-HT-secreting pNETs has been reported by Zandee et al. [16]. Despite liver metastases and urinary 5-HIAA levels increasing to up to 18 times the upper limit of normal, in our series only two out of seven patients presented with a carcinoid syndrome. Maurer et al. [8] showed similar results in a review of 29 cases of 5-HT-secreting “pancreatic carcinoids”; in these patients, no complete typical carcinoid syndrome occurred, despite the evidence of distant metastases in 69%, and elevated urinary 5-HIAA levels in 85% of cases. 

We can only speculate on the reasons why a high tumour burden and high levels of 5-HT metabolite (and thus of 5-HT secretion) are not always associated with the carcinoid syndrome: (1) no (or not enough, or with low affinity) 5-HT receptors are available in the target tissues (thus limiting its effects); (2) a hyperactivation of 5-HT clearance in specific tissue prevents any secondary effects of 5-HT. Moreover, some of the several factors and substances involved in the development of carcinoid syndrome may be lacking or inactive. In fact, diarrhoea and flushing in carcinoid syndrome may be due to a variety of tumour substances released, including 5-HT, tachykinins (substance P, neurokinin A and neuropeptide K) and prostaglandins [17], and several hypotheses have been proposed to explain the pathophysiology of these symptoms [18,19,20]. In our series, almost all patients showed a co-secretion of other hormones and substances detected in the serum, mostly CgA, NSE, calcitonin and gastrin. Zandee et al. [16] reported about 78% of patients with 5-HT-secreting pNETs with a serum CgA of more than 20 times the upper limit of normal, reflecting high tumour burden and a poor prognosis [16]. In addition, co-secretion of other ectopic hormones (i.e., calcitonin, gastrin and 5-HT) from an endocrine neoplasm may suggest a de-differentiation usually related to a more aggressive behaviour. 

Multihormonal immunostaining (including 5-HT) has been found in one third of all pNETs [21]; thus, it is a common finding without prognostic significance. In our series, even a G3 NET demonstrated positive immunostaining for 5-HT, chromogranin and also for calcitonin. Unfortunately, due to the small amount of tissue in biopsy samples in our series of unresectable tumours, we were unable to perform a complete immunohistochemical analysis in all patients, selecting those studies useful to define the neuroendocrine origin and the grade of the tumour. Serotonin-secreting pNETs may originate as de-differentiated tumours with a mixed cellularity, showing a co-secretion of other substances/hormones (i.e., 5-HT, calcitonin, gastrin, substance P, neurokinins, etc.), or from pancreatic enterochromaffin cells well differentiated in 5-HT production. In fact, small numbers of EC cells producing substance P, neurokinins, and 5-HT have been found in the human pancreas, scattered in the pancreatic duct and acini [22], and even the β-cells, as well as some other islet cell types, express all the genes required to synthesise, package, and secrete 5-HT [23]. This is in line with our findings, where all but one patient had a well-differentiated pNET. 

Non-functioning pNETs may show positive immunostaining for hormones, neuropeptides and amines, including 5-HT [3,4]. Serotonin-secreting pNETs have a very different prognosis from 5-HT-staining pNETs. The former (with or without associated carcinoid syndrome) are usually of large size at diagnosis and metastatic in up to 88% of cases [24], whereas 5-HT-staining pNETs are usually small, and patients can undergo surgery, providing the chance of a complete histologic and immunostaining study. In our series, no patient underwent a pancreatic resection, and all but one died from disease progression after a median time of 22.5 (range 5–158) months. 

Recently, using data from the SEER database, Dasari et al. [25] reported a median OS of 3.6 years for pancreatic NETs. Notably, G1–G2 pNETs with distant metastases diagnosed between 2000 and 2012 showed a median survival of 60 months, and 3- and 5-year survival rates were 62% and 50%, respectively [25]. In the present study, after multimodal treatment consisting mainly of chemotherapy, SS-A and/or ablation of liver metastases, patients demonstrated a 5-year disease-related survival rate of 42.9%, with an occasional long survival (up to 158 months). Zandee et al. [16] observed a 5-year survival rate of 46% in patients with 5-HT-secreting pNETs. Whether high levels of urinary 5-HIAA are related to a worse prognosis in pNETs or not is unknown, and the same topic is still a matter of debate in the more frequent small intestinal NETs [26]. It has been reported that the presence of a carcinoid syndrome is associated with a worse prognosis in pNETs [6]. In our experience, the two patients presenting with a carcinoid syndrome had the longest OS; one died of disease 158 months after diagnosis and another is still alive 117 months after diagnosis. 

In conclusion, 5-HT-secreting pancreatic NETs are rare entities, which include those tumours able to secrete high levels of 5-HT in the bloodstream, and consequently have high excretion of their urinary metabolite 5-HIAA. Although presenting with large pancreatic masses with liver metastases not amenable to resective surgery, patients complain of carcinoid syndrome in a minority of cases. This subset of pNETs are not associated with a worse prognosis than other stage IV pNETs reported in the literature; in fact, a five-year disease-related survival of 42.9% can be achieved with multimodal treatment.

## Figures and Tables

**Table 1:** Clinical presentation and laboratory tests at diagnosis in patients with serotonin-secreting pancreatic NETs.

No.	Obs	Gender/Age	Clinical Presentation	Carcinoid Syndrome	24-h Urinary 5-HIAA *	Serum 5-HT *	Other Serum NE Markersand GI Hormones	
							normal	elevated
1	1986	F/67	Abdominal pain	Yes (flushing, diarrhoea)	n.a.	5.1x	Gastrin, Glucagon, Calcitonin	NSE
2	1995	M/64	Asymptomatic	No	12.3x	3.8x	Gastrin	NSE, Calcitonin
3	1999	M/69	n.a.	n.a.	5.2x	n.a.	NSE, Insulin, Gastrin, Calcitonin	no
4	2002	M/44	Weight loss, dyspepsia	No diarrhoea	1.8x	n.a.	NSE, Gastrin	CgA, Glucagon, Calcitonin
5	2004	F/44	Cervical lymphadenopathy	No	6.7x	n.a.	NSE, Insulin	CgA
6	2010	F/38	Weight loss, jaundice, portal vein thrombosis, ascites, fatigue	Yes (flushing, diarrhoea)	17.4x	1.3x	NSE, SS, VIP, Calcitonin	CgA, Gastrin
7	2011	M/68	Abdominal pain, weight loss, fatigue	No diarrhoea	4.5x	2.1x	Gastrin	CgA, NSE, Calcitonin

Obs year of observation, F female, M male, n.a. not applicable, 5-HIAA 5-hydroxyndoleacetic acid, 5-HT 5-hydroxytryptamine, NE neuroendocrine, GI gastrointestinal, NSE neuron specific enolase, SS somatostatin, VIP vasoactive intestinal peptide, CgA chromogranin A. * Expressed as “times the upper limit of normal”.

**Table 2:** Pathological findings and follow-up in patients with serotonin-secreting pancreatic NETs.

No.	Pancreatic Site Size (cm)	Distant Metastases	Biopsy	TNM Stage [12]	NET/NEC Ki67	Immunohistochemistry		Other Therapies	Follow-Up (Months)	Status
						Positive	Negative			
1	Head 4.0	Bilobar liver	Liver	T2 Nx M1 IV	NET n.a.	5-HT 20–20–30% Grimelius	Insulin, Gastrin, PP	SS-A, CT ^a^	158	DOD
2	Tail 2.5	Bilobar liver	Liver	T2 Nx M1 IV	NET n.a.	CgA	n.a.	TACE	12	DOD
3	Body 4.0	Bilobar liver, mediastinal LN	Liver	T2 N1 M1 IV	NET n.a.	CgA, Grimelius	n.a.	CT ^b^	29	DOD
4	Tail 6.0	Bilobar liver	Abdominal LN	T3 N1 M1 IV	NET 3.4%	CgA, Syn, NSE	5-HT, Insulin, Gastrin, Glucagon, SS, PP, Calcitonin	SS-A, CT ^b^, TAE, PRRT	96	DOD
5	Body 10.0	Bilobar liver, cervical LN	Cervical LN	T4 N1 M1 IV	NEC n.a.	n.a.	n.a.	SS-A, CT ^c^	16	DOD
6	Body 4.0	Bilobar liver	Liver	T2 N1 M1 IV	NET 16%	CgA, Syn	n.a.	Biliary stent SS-A, CT ^d^ Liver MW Everolimus	117	AWD
7	Body 3.6	Bilobar liver	Liver	T2 Nx M1 IV	NET 70%	5-HT, CgA, Syn, Calcitonin	NSE, Insulin, Gastrin, Glucagon, SS, PP, VIP	CT ^e^	5	DOD

LN lymph node, NET neuroendocrine tumour, NEC neuroendocrine carcinoma, n.a. not applicable, 5-HT 5-hydroxytryptamine, CgA chromogranin A, Syn synaptophysin, NSE neuron specific enolase, PP pancreatic polypeptide, SS somatostatin, VIP vasoactive intestinal peptide, SS-A somatostatin analogue, CT chemotherapy, TACE transarterial chemoembolisation, TAE transarterial embolisation, PRRT peptide receptor radionuclide therapy, MW microwave, DOD died of disease, AWD alive with disease. ^a^ Dacarbazine. ^b^ not available. ^c^ First line: paclitaxel, cisplatin, and gemcitabine; second line: doxorubicin and streptozotocin. ^d^ First line: 5-fluorouracil, dacarbazine, and epirubicin; second line: capecitabine. ^e^ Epirubicin, 5-fluorouracil and dacarbazine.
